# Anchylosis of the Jaw

**Published:** 1849-01

**Authors:** A.C. Castle


From the "Boston Medical and Surgical Journal."

### ANCHYLOSIS OF THE JAW OF THIRTY-FOUR YEARS DURATION.

The following interesting case we copy from the above well-known periodical:

Mr. ——, a native of Scotland, aged 50 years. At the age of 11 years he was apprenticed to the merchants' marine, from a Scottish port. Whilst on the homeward-bound voyage from the West Indies, in 1813, the vessel on which he was aboard was pursued by a French frigate. The chase was long and exciting, and every effort to increase the speed of the vessel, to effect an escape, was of course made by the captain. For this purpose, various practicable alterations were effected in the vessel's trim, and every "stitch" of canvas that could be brought to bear, was put into requisition. The lad was sent aloft to "loose" the "main-royal sail," when from a heavy plunge the ship made in a high running "head sea," and a squall of wind simultaneously striking her, the "royal" mast was "carried away," i. e., snapped asunder, and precipitated him "in-board" of the long-boat below; breaking both his legs—one a compound fracture of the fibula and tibia, midway between the ankle and knee, and the other a compound fracture at the knee-joint, driving the *patella* almost through the joint. Both arms were broken, and three ribs on the left side, and the left *clavicle.* The teeth were forced through the lips, in consequence of his face striking against the inside of the boat; but no other injury was inflicted on the head, otherwise than that of insensibility arising from the general concussion and shock received by the sensorium and nervous system. The vessel escaped from the grasp of her pursuer, and in the absence of better assistance, the ship's carpenter, for the nonce, doffed his office of surgeon to "*sprung*" masts and yards, solutions of continuity in seams, and strictures in the pump's urethra, and devoted his skill to carpentering fractured bones, and "fishing" the broken limbs of the unfortunate lad, and thus with "home-made" rough "splints"

he placed the fractured limbs in position, first having removed, with his chisel! the comminuted portions of fractured bones, and, to the best of his abilities, bringing together the flesh by the adhesive qualities of plaster comprised of tar-pitch and canvas. He then proceeded from the ship's "medicine chest," and strict diet, to place him under rigid "antiphlogistic" treatment, and he was in every way—using his own words—"well cared for."

Several days after, the vessel made the port of Portsmouth, Eng., and he was taken ashore, to the marine hospital. The limb, implicated with the compound fracture at the knee-joint, was amputated between the middle and lower third of the thigh; and the other fractures treated *secundem artem*, with the view, if possible, of ultimately saving them. So absorbed were the surgeons with the numerous fractures, and the desire to save his life, that they altogether lost sight of its important portal, the mouth, further than bringing together and preserving in apposition the wounds of the lips. The muscles and ligaments of the jaw daily contracted, but so gradually that even the patient himself, overwhelmed as he was with sufferings, *did not perceive* the advance of his terrible affliction, until he discovered that he could not, upon the return of his appetite, open his mouth to partake of food. His jaws were firmly fixed, and the teeth closely locked upon each other. Treatment and experiment alike failed to overcome this additional calamity, and he was, in due course of time, in all other respects, "discharged cured."

After the war he emigrated to this country, and for many years has been a resident of this city. Several years since, on the eve of Dr. Mott's departure for Europe, he consulted this gentleman as to the possibility of obtaining relief by an operation. Nothing was done, and tumefaction and suppuration supervened. He was, in consequence of the successful operation which I had performed, in replacing, by artificial means, the loss of large portion of the maxillary bones and teeth (from a gunshot wound) in the case of Lieut. S., U. S. N., advised to consult me with regard to any chance of success attending an operation for securing "artificial joints" to the inferior maxillary bone.

Having examined the case, I advised him by no means to submit to an operation of this kind; that such an operation had been performed several years since, by an eminent surgeon, upon the jaw of a young lady* similarly affected, with the most disastrous results; that she had applied to me for relief, and the only solace she ever received for her sufferings, was the overcoming the frightful deformity of an *absent cheek*, which was successfully and beautifully accomplished with an artificial one made by my friend Dr. Smilie, dentist, of this city. The maxillary bones, gums and teeth on one side, were completely exposed, her jaws locked, and the teeth rigidly closed and fixed together, thus presenting the appearance of a grinning skeleton, and on the other exhibiting all that was youthful, healthful, and beautiful. The failure of youthful resources to secure artificial joints, did not in this instance warrant a similar experiment upon those of age.

I found his teeth, as already stated, firmly locked together; the gums tumefied and vegetating, pus oozing from their edges and the alveolar dental periosteum, the breath feverish and fetid. I proceeded to remove the several teeth after their order, two superior and two inferior posterior *bicuspids* on either side, four superior and four inferior *multicuspids*, and the two superior lateral *incisors*—the two central teeth having originally been extracted for the purpose of enabling him to speak and partake of "spoon meats," upon which he has subsisted for thirty-five years.

* The anchylosis affecting the maxillary bone of this lady was superinduced from the cauterizing the inner surface of the mouth during her illness (fever, cancrum oris.) The surface sloughed, and produced an angry ulcer. The cauterizing was continued, and the consequences were, that the whole of the left cheek was corroded entirely away from its junctions with the maxillary bones, and the attempt of an eminent dentist, with her surgeon, to extract a molar tooth, fractured her jaw, which, in connection with the disease, was the remote and exciting cause of the anchylosis. The operation for producing artificial joints, was performed by cutting through the ascending portions of the jaw, just below where the condyles and coronoid processes bifurcate. From the account of the case given by the young lady, I could not rightly comprehend what treatment she had received, or what cause was assigned for the failure.

I left in the mouth two superior and two inferior *cuspidati*, the same number of *bicuspids*, the four inferior *incisors*, and the four *sapientiæ* teeth. The removal of the above teeth gave him great relief. It enabled him to cleanse his mouth upon the inside and outside of the teeth; it gave ample room to the tongue, both for speaking and crushing the food, that he was in the habit of taking, against the roof of the mouth, and, to his astonishment, improved the sense of taste to such an extent that he conceived—until explained to him that closed jaws materially affected this sense—that the "character of his food had changed." The tumefaction and suppuration of the gums and periosteum subsided, as did also the fetid odor of the breath, and the digestive powers recovered from the prostration superinduced by these exciting causes. He was most anxious, at the time, that I should extract *all* the teeth from his head, particularly the lower incisors, that he might enjoy still *more* space in his mouth for his "cribb'd" tongue. I should have complied with his desire, but that the position in which his jaws were fixed, and the *tout ensemble* in which the maxillary apparatus was placed, and prognosis, did not indicate nor warrant such a mode of procedure. It is a well-demonstrated fact, that the inferior maxillary bone undergoes various changes, more so than any other bone of the human frame. At birth, the angle of the jaw is obtuse; at maturity, when all the teeth are developed, it forms a right angle; and as the teeth make their appearance, and are severally lost to the animal economy, so does the jaw undergo material physical change, either with the addition, or from the loss of each tooth. I prognosticated, therefore, that should I extract *all* the teeth, the muscular contraction would continue, and however firmly the *condyles* of the jaw might be anchylosed with their glenoid cavities, that the immense power of the maxillary muscles, and the constant strains upon them, would draw the *ramus* of the jaw upon the superior maxillary bone, and anteriorly close the jaws altogether, so as to deprive him of the ordinary functions of the lips (from their compression upon each other) and the mouth; and I did not extract the lower incisors, because it would have deprived him of this

"dam," as it were, to retain the saliva, as well as prevented the forming of the *dento-lingual* articulations of the voice.

His second visit to me proved the fortunate prognosis that I had made upon his case. The muscles *had* contracted so as to force the remaining teeth, which I had left in the mouth, for the purpose of keeping the jaws apart, considerably out of their perpendicular lines of position. The dentes sapientiæ were now decayed, and their spiculated surfaces were not only forced into each other, but were also forced over the *mylo-hyoidean* line of the jaw into the throat, contracting its capacity, and pressing down the tongue and the tonsils, and thus materially affecting the function of deglutition. Tumefaction and considerable inflammation of the gums and palate were present, the pendulum of which was elongated and exceedingly troublesome. He was much depressed in spirits, feverish, and complained of neuralgic pains over his temples, in the "balls" of his eyes, thence to the back of his head, down his arms, and to various parts of his body, and was oppressed with the feeling that he must die of "lock-jaw," or suffocation. "One of these times, I shall give one struggle, and it will be all over with me," were his desponding remarks. He having great confidence in me, my encouraging explanations that *tetanus* and his "lock-jaw" were totally and vitally different, relieved his mind and feelings from the oppression that was weighing heavily upon them. I then extracted the four "wisdom teeth," and one inferior canine tooth, which had been forced *forward*, out of its line of position, by the pressure upon it. I snipped off considerable portions of the tumefied and vegetating gums* near the tonsils, and applied powerful astringent washes to the mouth, and administered alteratives internally. His natural "spirits" have recovered their buoyancy, and his difficulties (for the present) are at an end—expressing himself yesterday, when I dismissed him, as "altogether another man."

Should the contraction of the maxillary muscles continue so as to draw still upon the jaw, I shall endeavor to overcome the

* I did wrong to snip off the vegetating gums. It was the cause of much trouble both to myself and patient.

difficulty and prevent it, by forming a wedge of gold upon either side of the mouth, in the shape of an hour-glass, the ends, of course, to be nicely adapted to the gums on the dental surface of the maxillary bones, somewhat after this manner,  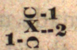 No. 1, 1, representing the ends of No. 2, letter X, or hour-glass, to be placed perpendicularly between the superior and inferior maxillary bones; by which means I should hope to keep them apart, without local or constitutional irritation.

A. C. CASTLE, M. D.,
New York, May 19, 1848. Surgeon Dentist.

